# Career Development of Academic Staff in Faculties of Dentistry by Means of Mentorship Programs: Protocol for a Scoping Review

**DOI:** 10.2196/27239

**Published:** 2021-07-21

**Authors:** Seyi Amosun, Faheema Kimmie-Dhansay, Greta Geerts, Reneda Basson

**Affiliations:** 1 Faculty of Dentistry University of the Western Cape Cape Town South Africa

**Keywords:** scoping review protocol, academic staff development, mentorship, capacity development, dental education, dentistry, dental educators

## Abstract

**Background:**

Globally, the demands on dental educators continue to diversify and expand. Due to their importance and value, mentoring programs have been acknowledged as a means of recruiting, developing, and retaining academics in dental education.

**Objective:**

This protocol is for a scoping review that aims to identify the goals of mentoring programs for academic staff in dental faculties and determine how these programs were structured, delivered, and evaluated.

**Methods:**

The review will be performed in accordance with the Joanna Briggs Institute’s methodology for scoping reviews, which covers both qualitative and quantitative scientific literature as well as grey literature written in English and published between 2000 and 2020. The databases will include PubMed, Ovid, the Educational Resources Information Center database, Science Direct, Scopus, Google Scholar, Trove, Web of Science, Openthesis.org, and the website of the American Dental Education Association. A manual search will also be conducted by using the reference lists of included studies to identify additional articles. Working independently, the authors will participate iteratively in literature screening, paper selection, and data extraction. Disagreements between the reviewers will be resolved by discussion until a consensus is reached or after consultation with the research team. Key information that is relevant to the review questions will be extracted from the selected articles and imported into a Microsoft Excel file. The PRISMA-ScR (Preferred Reporting Items for Systematic Reviews and Meta-Analyses Extension for Scoping Reviews) will be used to guide the reporting of this protocol.

**Results:**

The search for appropriate literature has commenced, and we aim to present the results before the end of the 2021 academic year.

**Conclusions:**

The development of formal mentorship programs for academics in dental education will enhance the retention of academic staff.

**International Registered Report Identifier (IRRID):**

PRR1-10.2196/27239

## Introduction

Mentoring was first developed in the United States of America in the 1970s within large corporations to support junior staff [1] but has now extended to other contexts, including the university-based education platforms of health professionals [2-13]. Over the years, the reforms in the education of health professionals have contributed to the doubling of life expectancy during the 20th century [4]. However, in the beginning of the 21st century, health advances were not being shared equitably due to gaps and inequalities in health education that existed between and within countries. To advance opportunities for health equity within and between countries, a call for redesigning the curricula of health professionals was made. Instructional and institutional reforms were proposed, and these would be guided by two outcomes—transformative learning and interdependence in education [5]. As transformative learning is considered an outcome of instructional reforms, specific recommendations were made, such as the strengthening of educational resources with special emphasis on the capacity development of faculty and academic staff, including those of medical schools in Africa. Through the provision of rewarding and stable career trajectories and performance-based constructive assessment, the need to increase investments in the education of educators was highlighted.

Mentoring is difficult to define, but the Standing Committee on Postgraduate Medical and Dental Education described it as a process “whereby an experienced, highly regarded, empathetic person (the mentor) guides another (usually younger) individual (the mentee) in the development and re-examination of their own ideas, learning, and personal and professional development. The mentor, who often works in the same field as the mentee, achieves this by listening or talking in confidence to the mentee” [14]. Although mentoring is considered to be central to education in academic medicine and other health professions, it is inhibited by the increased number of clinical, administrative, research, and other educational demands on academics, thus reducing the time they need to devote to a mentoring relationship [11-14]. Unfortunately, the unique challenges encountered by women in academics have not yet been satisfactorily addressed [1,5,8,12]. The absence of mentorship has often resulted in a failure to retain junior academics in higher education institutions or within academia altogether.

It is helpful to gain some insight into the global context of dental education to appreciate the need for mentorship programs that enhance the careers of academic staff who are preparing for the future generation of oral health care professionals. It has been estimated that under 3 to 5 billion people across the world are affected by oral diseases [15]. The World Health Organization has therefore focused on increasing the awareness of oral health worldwide, as it is considered to be an important component of people’s general health and quality of life. Unfortunately, oral disease is still a major public health problem in high- and low-income countries, and the burden of oral disease is growing in many low‐ and middle-income countries [16,17]. Untreated oral disease is a visible reminder of existing health inequalities, and ensuring access to adequately trained oral health professionals has become critical [18].

The following two approaches have influenced the practice of dentistry globally: the odontology and stomatology models [19-21]. Odontology is the study of oral disease and disorders of the oral cavity, including their diagnosis, treatment, and prevention. Stomatology is the study of the mouth, and it connects general medical health and disease with diseases of the mouth [21]. The duration of dental education across the world ranges from 5 to 9 years [20]. Unfortunately, there are limited details about dental education in Africa despite the oral health challenges in this continent [22-24] and in other countries where inequities in access to oral care are also evident [25]. The presumption that colonization influences dental education in Africa and other places could have contributed to the calls for the decolonization, indigenization, and transformation of oral dental education [26-28].

In light of the recommendation to transform the education of health professionals in order to strengthen health systems in the 21st century [5], professional bodies like the American Dental Education Association have made calls for major changes in the training of oral health professionals [26-31]. A 2-phase project named “Advancing Dental Education in the 21st century” is a current driver of change in the United States of America. The project aims to develop practical strategies that dental and allied dental educational institutions can implement to address long-range challenges related to finances, education, scholarship, diversity, and the need to change disease and practice trends by 2040 [32-36]. Similarly, a partnership between the American Dental Education Association and the Association for Dental Education in Europe, which have strong historical relationships, led to the creation of another project named “Shaping the future of Dental Education” [37-41]. The first meeting between the two associations was attended by over 180 participants who represented 39 countries and sought to explore the roles and capabilities of dental educators in impacting global dental health education.

Curriculum changes were recommended by the two projects to prepare oral health professionals to address more diverse, multicultural patient populations; older patients with dental needs and comorbidities; younger patients with fewer restorative and prosthodontic treatment needs; and vulnerable, disadvantaged populations with unmet needs [32-41]. These professionals would require skills for serving as good team members and team leaders of intra- and interprofessional teams as well as skills in communication, collaboration, supervision, critical thinking, and problem solving. These professionals would also need to understand scientific methods, evaluate research findings, and decide what to incorporate into patient care. Additional factors that would impact dental education in preparing oral health professionals for 2040 and beyond would include skills for addressing the social determinants of health [36] and adapting to different teaching and learning methodologies [41,42]. To achieve the goals of the change processes that were set out for the 21st century, the capacity development of academic staff was considered to be essential for their recruitment and retention. This was in line with earlier recommendations of the American Dental Education Association [5,35,43,44].

The quality of academics and their willingness to lead the transformative changes in dental education have become essential. Due to having little to no previous experience and coming from diverse backgrounds, novice academics can readily feel overwhelmed, unsupported, and discouraged in their attempts to meet the demands of success in academics [6,7,26]. Therefore, the objective of this paper is to present a protocol for a scoping review that aims to map out literature and produce an evidence-based synthesis of data regarding the mentorship of academic staff in dental faculties.

## Methods

### Summary of the Scoping Review

The overall objective of the proposed scoping review is to explore formal and informal mentorship programs in dental faculties that are designed to develop academic staff members’ careers. The review will be conducted in accordance with the Joanna Briggs Institute (JBI) framework for scoping reviews, which acknowledges the following six methodological stages: (1) the identification of the research question; (2) the identification of relevant studies; (3) the selection of studies; (4) the extraction and charting of the results; (5) the collation, summarization, and reporting of results; and (6) consultation with stakeholders (optional) [45].

### Identifying the Research Questions

The proposed scoping review aims to answer the following three questions: within existing literature, (1) what were the goals of mentoring programs for academic staff in dental faculties, (2) how were these programs structured and delivered, and (3) how were the programs evaluated?

### Identification of Relevant Studies

#### Search Strategy

In consultation with the librarian of the Faculty of Dentistry at the University Western Cape, the electronic search for literature will focus on retrieving articles that were published in peer-reviewed journals between 2000 and 2020 via a systematic search of the following bibliographic databases: PubMed, Ovid, the Educational Resources Information Center database, Science Direct, Scopus, Google Scholar, Trove, Web of Science, Openthesis.org, and the website of the American Dental Education Association [46]. In addition, a manual search will be conducted by using the reference lists of included studies to identify additional articles. Articles in grey literature that are considered to be relevant to the research questions and objectives will also be included, and if this is done, the information sources will be reported. Only articles written in English will be considered.

The 3-step search strategy, which is explained in the JBI Reviewer’s Manual, was followed [45]. The first step involved consulting a librarian in the Faculty of Dentistry. The librarian conducted pilot searches by searching for the terms *mentor*, *training*, and *dental* in the abstracts of relevant articles and grey literature from a predefined list and tested the search strategy with different databases. Articles published before the year 2000 often conflated mentoring with supervision, role modelling, coaching, advising, networking, and sponsorship [5,8,12]. Based on the outcome of the pilot searches (Table 1), we determined the search strategy that will be used when the scoping review is conducted. With the assistance of the faculty librarian, the search strategy was drafted and further refined through team discussion.

**Table 1:** Search strategy for PubMed. The search was conducted on April 7, 2021.^a^

Search number	Query terms	Records retrieved, n
1	*Training*	1,283,644
2	*Mentorship OR mentor*	16,936
3	*Dental OR dentistry*	479,241
4	*Training AND mentorship OR mentor AND dental OR dentistry*	574

^a^The search was limited to literature published from 2000 to 2020, those written in English only, and those that involved human subjects only.

#### Selection of Studies

Two reviewers (SLA and FKD) will conduct searches in all identified databases, and all search results will be imported into Rayyan Qatar Computing Research Institute software to ensure that a systematic and comprehensive search is performed and to document the selection process [47]. Working independently, the same reviewers will manage Rayyan and review the titles and abstracts to identify and remove duplicate citations. If necessary, full-text copies of the short-listed studies will be obtained to find more details. The reviewers will consider studies that focused on the mentoring of academic staff in the dental faculties of the following departments and specialties: orthodontics, maxillofacial and oral surgery, prosthodontics, periodontics, pediatric dentistry, maxillofacial and oral radiology, oral pathology, oral forensic pathology, clinicians, specialists, oral hygienists, and dental therapists. However, the considered studies will not be limited to these topics. Studies that focus on allied health specialties (dietetics, nursing, occupational therapy, physiotherapy, psychology, chiropractic, midwifery, social work, etc) and nondental professionals (medical professionals and veterinary science professionals) and studies that do not fit into the conceptual framework of the proposed scoping review will be excluded.

The proposed scoping review will consider both experimental and quasi-experimental study designs, including randomized controlled trials, nonrandomized controlled trials, before-and-after studies, and interrupted time series studies. In addition, analytical observational studies, including prospective and retrospective cohort studies, case-control studies, and analytical cross-sectional studies, will be considered for inclusion. This review will also consider descriptive observational study designs, including case series, individual case reports, and descriptive cross-sectional studies, for inclusion. Qualitative studies that focus on but are not limited to qualitative data, including phenomenology data, grounded theory, ethnography data, qualitative descriptions, action research, and feminist research, will also be considered. In addition, systematic reviews and texts and opinion papers that meet the inclusion criteria will also be considered for inclusion. All references from identified grey reports will be hand-reviewed to determine if the literature is appropriate for inclusion in the scoping review.

Any disagreements between the two reviewers will be resolved by discussion until a consensus is reached or after consultation with the research team. Once a decision is reached on the final list of selected articles, the full texts of the articles will be accessed. The reference management software Mendeley Desktop for Windows will be used to store, organize, cite, and manage all selected references [48]. Two reviewers (SLA and FKD) will independently review the articles to determine whether they meet the inclusion criteria. Disagreements regarding inclusion will again be discussed and resolved by consensus among the reviewers or with the other two authors (RB and GG). A PRISMA-ScR (Preferred Reporting Items for Systematic Reviews and Meta-Analyses Extension for Scoping Reviews) flow diagram will be used to delineate the search decision process for the scoping review [49]. The full texts of selected citations will then be independently assessed by two reviewers (SLA and FKD) in detail against the inclusion criteria. Reasons for the exclusion of full-text studies that do not meet the inclusion criteria will be recorded and reported in the scoping review. Again, any disagreements that may arise between the two reviewers will be resolved via consultation with the other two authors (RB and GG) to reach a consensus.

### Extracting and Charting the Results

A Microsoft Excel file will be developed by the two reviewers (SLA and FKD) and pilot tested to determine whether it satisfactorily captures information that is consistent with the purpose and questions of the scoping review. General information that will be included in the Excel chart will include a description of study characteristics—the year of publication, the country of origin, the geographical location in which the research was conducted, study aims and purposes, the methodology and sample size, and descriptions of study populations. Key information that is relevant to the review questions will be independently extracted from the selected articles by the two reviewers and imported into the Microsoft Excel file. This will be an iterative process conducted by the two reviewers, and the file will be continuously updated.

### Data Availability

The data generated for this scoping review protocol will be made available upon reasonable request.

## Results

The fifth stage of the JBI framework for scoping reviews is collating, summarizing, and reporting the results. The PRISMA-ScR [49] will be used to guide the reporting of the scoping review protocol and will subsequently be used to structure the reporting of the full review.

The search for appropriate literature has commenced, though it has been delayed due to the sudden death of the librarian during the peak of the COVID-19 pandemic. However, we aim to present the results before the end of the 2021 academic year.

## Discussion

The sixth stage of the JBI framework for scoping reviews (consultation with stakeholders) is incorporated into this section. The prevalence of oral diseases across the world is still a major concern, and there are calls for additional oral health professionals to improve access to care [15-17]. The two change projects that are referred to in this paper [32-42] provide a framework for the training of oral health professionals during the 21st century that is in line with the recommendations of the Lancet Commission [5]. Higher education institutions cannot ignore these transformational drivers of change in dental education. Upon entering a career in academia, there is the expectation that novice academics can meet the increasing demands of teaching, scholarly activity, and research; perform administrative tasks; and participate in both university and community services [7]. Oral and dental care play an important role in the health and well-being of the population. Further, mentorship will help mentees to fulfill their career aspirations and will contribute to meeting the oral health needs of the population. Therefore, the purpose of the proposed review is to identify the goals of mentoring programs for academics in dental faculties and determine how these programs were structured, delivered, and evaluated**.** As this review will only include data that are already in the public domain, ethics approval will not be sought. The data generated from the scoping review will be accessible upon request, as the library of the University of Western Cape is developing a data management plan for storing and sharing research data securely on the university’s research data repository [50]. However, the articles selected for review will be selected regardless of methodological quality and the risk of bias, as analyzing these factors is outside of the scope of the review. This may be a limitation of the study.

## Abbreviations

JBI: Joanna Briggs Institute
PRISMA-ScR: Preferred Reporting Items for Systematic Reviews and Meta-Analyses Extension for Scoping Reviews
